# Interferon beta treatment is a potent and targeted epigenetic modifier in multiple sclerosis

**DOI:** 10.3389/fimmu.2023.1162796

**Published:** 2023-05-30

**Authors:** Alexandre Xavier, Maria Pia Campagna, Vicki E. Maltby, Trevor Kilpatrick, Bruce V. Taylor, Helmut Butzkueven, Anne-Louise Ponsonby, Rodney J. Scott, Vilija G. Jokubaitis, Rodney A. Lea, Jeannette Lechner-Scott

**Affiliations:** ^1^ School of Biomedical Science and Pharmacy, University of Newcastle, Newcastle, NSW, Australia; ^2^ Department of Neuroscience, Central Clinical School, Monash University, Melbourne, VIC, Australia; ^3^ Hunter Medical Research Institute, Immune Health research program, Newcastle, NSW, Australia; ^4^ Department of Neurology, John Hunter Hospital, Newcastle, NSW, Australia; ^5^ School of Medicine and Public Health, University of Newcastle, Newcastle, NSW, Australia; ^6^ Florey Institute of Neuroscience and Mental Health, The University of Melbourne, Parkville, VIC, Australia; ^7^ Menzies Institute for Medical Research, University of Tasmania, Hobart, TAS, Australia; ^8^ Neuro-Immunology Registry, MSBase Foundation, Melbourne, VIC, Australia; ^9^ New South Wales (NSW) Health Pathology, John Hunter Hospital, Newcastle, NSW, Australia; ^10^ Centre of Genomics and Personalised Health, School of Biomedical Sciences, Queensland University of Technology, Kelvin Grove, QLD, Australia

**Keywords:** multiple sclerosis, methylation, interferon beta (IFN beta), disease modifying therapy (DMT), inflammation, epigenetics (DNA methylation)

## Abstract

**Introduction:**

Multiple Sclerosis (MS) has a complex pathophysiology that involves genetic and environmental factors. DNA methylation (DNAm) is one epigenetic mechanism that can reversibly modulate gene expression. Cell specific DNAm changes have been associated with MS, and some MS therapies such as dimethyl fumarate can influence DNAm. Interferon Beta (IFNβ), was one of the first disease modifying therapies in multiple sclerosis (MS). However, how IFNβ reduces disease burden in MS is not fully understood and little is known about the precise effect of IFNβ treatment on methylation.

**Methods:**

The objective of this study was to determine the changes in DNAm associated with INFβ use, using methylation arrays and statistical deconvolutions on two separate datasets (total n_treated_ = 64, n_untreated_ = 285).

**Results:**

We show that IFNβ treatment in people with MS modifies the methylation profile of interferon response genes in a strong, targeted, and reproducible manner. Using these identified methylation differences, we constructed a methylation treatment score (MTS) that is an accurate discriminator between untreated and treated patients (Area under the curve = 0.83). This MTS is time-sensitive and in consistent with previously identified IFNβ treatment therapeutic lag. This suggests that methylation changes are required for treatment efficacy. Overrepresentation analysis found that IFNβ treatment recruits the endogenous anti-viral molecular machinery. Finally, statistical deconvolution revealed that dendritic cells and regulatory CD4+ T cells were most affected by IFNβ induced methylation changes.

**Discussion:**

In conclusion, our study shows that IFNβ treatment is a potent and targeted epigenetic modifier in multiple sclerosis.

## Introduction

Interferon beta (IFNβ) was the first disease-modifying therapy (DMT) approved for use in multiple sclerosis (MS). Although it has been superseded by higher efficacy treatments in the last decade, it remains as a first-line treatment for MS in many countries. Despite its widespread use since 1995, the full biological mechanism of IFNβ treatment remains unclear as not all molecular targets have been identified. In short, IFNβ binds to the ubiquitously expressed interferon alpha/beta receptor (1), ultimately leading to suppressed inflammation (2) through various downstream events such as prevention of the blood–brain barrier migration (3), reduction in T cell activation, or promotion of oligodendrocyte differentiation [Hojati et al. (4)].

DNA methylation (DNAm) is a reversible epigenetic mechanism where a methyl group is added to a cytosine located next to a guanine, also known as a CpG. DNAm has been linked to gene expression modulation and is affected by both genetics and environment (5, 6). Epigenome-wide association studies (EWASs) of blood cell DNA have revealed that epigenetic signatures can change with various disease pharmacotherapies such as dimethyl fumarate (7), corticosteroids (8), or chemotherapies (9). IFNβ treatment has been shown to modulate global methylation in small studies using DNA from whole blood (10) and monocytes (11) in people with MS (pwMS). However, no specific description of those changes has been reported at the gene level. Analysis of DNAm may provide insight into precise mechanism of action of IFNβ by identifying new potential downstream target genes.

## Methods

This study used two different existing datasets (see **Table 1** for details): a discovery set (12) and a replication set (13). Each dataset was composed of pwMS, either untreated or treated with only IFNβ at the time of blood collection. DNA was extracted from whole blood and bisulfite-converted before being used on methylation arrays.

**Table 1:** Participant demographics.

	Discovery study		Replication study	
	Treated with IFNβ	No IFNβ treatment	Treated with IFNβ	No IFNβ treatment
**N**	31	83	33	202
**Age (mean years ± SD)**	47.5 ± 10.6	52.1 ± 10.3	39.2 ± 10.8	38.6 ± 9.75
**Gender (% Female)**	100	100	72.7	76.2
Relevant Metrics				
**MS stage (CIS/RR/SP/PP)**	0/25/6/0	0/40/43/0	1/29/0/3	119/71/0/12
**Treatment (more details)**	Betaferon (1b): 12 Avonex (1a): 7 Plegridy: 2 Rebif 44 (1a): 10	N/A	Betaferon (1b): 19 Avonex (1a): 6 Rebif 44 (1a): 8	N/A
**Avg. age of onset (mean ± SD)**	40.1 ± 1.63*	35.8 ± 1.18*	39.2 ± 10.81	38.59 ± 9.74
**Avg. disease duration in years (mean ± SD)**	8.3 ± 1.07*	16.3 ± 1.13*	1.57 ± 1.12	1.27 ± 2.16
**EDSS closest to collection (mean ± SD)**	2.3 ± 0.47	4.6 ± 0.35	1.65 ± 1.47	1.61 ± 1.47

*Two samples missing diagnosis date.

### Patient recruitment and blood collection

The discovery dataset consisted of samples from Australians attending outpatient clinics at the Royal Melbourne Hospital (VIC), Alfred Health (VIC), John Hunter Hospital (NSW), and Flinders Medical Centre (SA), who were participating in the MSBase Registry (14) and were recruited as part of the severity genome-wide association study (GWAS) (15). This dataset was composed of 114 female patients with MS, 31 of whom were treated with IFNβ and 83 of whom were untreated. There was a mix of relapsing and progressive phenotypes.

The replication dataset was derived from the Ausimmune study (12), a multi-center Australian study. The replication dataset consisted of 33 IFNβ-treated pwMS (24 female and nine male patients) and 202 untreated pwMS (154 female and 48 male patients). All participants in the Ausimmune study were recruited after their first clinical evidence of demyelination. Demographic details for both groups are listed in **Table 1**.

### DNA extraction

DNA was extracted from whole blood using the QIAamp DNA Blood Mini Kit ™, The Netherlands. Extracted DNA was quantified using the Qbit (Invitrogen™, USA), and integrity was assessed using the genomic DNA tapes on a TapeStation (Agilent™, USA) using the DNA integrity number (DIN) as a metric. All samples had a DIN ≥ 7, with minimal genomic DNA degradation.

### Methylation arrays

Genomic DNA (500 ng) was bisulfite-converted using the EZ- DNA Methylation™ Kit (Zymo) according to manufacturer’s converted DNA was hybridized to the Illumina Infinium Methylation 850K EPIC BeadChip arrays (hereafter referred to as EPIC arrays). To avoid batch effects, samples were randomized on the EPIC arrays using the OSAT R package (16). Arrays were read using the iScan (Illumina™) to produce raw Idat files.{Campagna, 2022 #259}

### Genotyping arrays

We hybridized 200 ng of genomic DNA to the Illumina Global Screening Array (hereafter referred as GSA) and processed according to manufacturer’s protocol. To avoid batch effects, samples were randomized on the GSA chips using the OSAT R package (16). Arrays were read using the iScan (Illumina™) to produce raw Idat files.

### Statistical analysis

Analysis of EWAS data was performed using the ChAMP R package (17, 18). To summarize, Idat files were loaded and filtered to remove low-performing probes, probes mapping to multiple loci, and low-performing samples. Probes next to known polymorphisms and on XY chromosome were retained. Beta values were normalized using the Beta-Mixture Quantile (BMIQ) method (19). Batch effects on both array and chip levels were corrected using the ComBat algorithm (20).

The final model used to identify differentially methylation positions (DMPs) was constructed using logistic regression, whereby phenotype (IFNβ treated or untreated) was considered the outcome variable, and each CpG beta value and cell-type proportions [for natural killer (NK) cells, monocytes, lymphocytes B, CD8^+^ T cells, CD4^+^ T cells, and neutrophils] were considered predictors. For CpG *i*,


$$glm(Outcome_{\frac{Treated}{Untreated}}\;\sim \;CpG_{i}+Age+Sex+CellFraction)$$

The mean difference in methylation between IFNβ-treated and untreated group was used to assess the effect size [i.e., delta beta (Δβ)]. When combining both studies, we used the following model:


$$glm(Outcome_{\frac{Treated}{Untreated}}\;\sim \;CpG_{i}+Age+Sex+CellFraction+Dataset_{\frac{Discovery}{Replication}})$$

where $Dataset_{\frac{Discovery}{Replication}}$ represents the study of origin (replication or discovery).

When possible, we used a single CpG or index CpG to represent a cluster of CpGs that had a Δβ in the same direction and that mapped to a single gene. Methylation treatment score (MTS) was constructed as follows using every significant index DMP identified in the discovery study that was also replicated in the validation study:


$${\text{MTS}}_{sample}=\;\sum^{}\beta value_{Sample\;at\;CpGi}\;\times \;\Delta \beta_{CpGi}$$

where ${\text{MTS}}_{sample}\;$ is the MTS for a specific sample, $\beta value_{Sample\;at\;CpGi}\;$ is the beta value at index CpG_i_ used to construct the MTS, and $\Delta \beta_{CpGi}\;$ is the weight used (i.e., the average Δβ identified for CpG_i_ between treated and untreated samples in the discovery cohort). We used the calculated MTS to create a linear model evaluating the relationship between MTS and the time since first treatment/injection:


$$lm\left(\text{MTS}\;\sim \;log_{10}\left(Days\;since\;first\;treatment\right)\right)$$

Immune cell proportions were calculated using the methylResolver R package (21). Cell-specific analysis was done using a linear regression model for each cell type (either monocytes, B cells, NK cells, neutrophils, CD4^+^ T cells, or CD8^+^ T cells). For each cell, we constructed the following model for every DMP identified in the combined dataset:


$$lm(CpG_{i\;}\sim \;CellProp+\left(CellProp\times \;Treatment_{0-1}\right)+Sex+Age+Cohort)$$

where $CpG_{i\;}$ is the CpG of interest; CellProp is the cell proportion between 0 and 1; sex, age, and dataset of origin (replication or discovery) were used as covariates. $CellProp\times \;Treatment_{0-1}$ represents the interaction term between cell proportion and treatment status and was the variable of interest. We removed any CpG with unadjusted significance under 9.8 × 10^−8^ (genome-wide significance) or with an absolute beta coefficient under two from the constructed linear model.

### Overrepresentation analysis

Overrepresentation analysis (ORA) was performed using the R package clusterprofiler (22), with the biological process annotation and a background of all genes present on the EPIC methylation array (n = 26,650). The list of gene symbols used as input is as follows: IFI44L, IFI44, ADAR, RABGAP1L, CMPK2, RSAD2, IFIH1, SP100, PARP9, PARP14, PLSCR1, TNK2, DDX60, TAP1, TNRC18, PDE7A, LY6E, TRIM14, IFIT3, IFIT1, IFITM1, IRF7, TRIM22, PARP11, OAS1, OAS2, OASL, EPSTI1, IRF9, IFI27, B2M, BISPR, PRIC285, MX1, USP18, and ODF3B.

### Genome-wide association study

We first identified the individuals within the top and bottom quartiles based on MTS (each group, n = 16; bottom quartile, MTS< −0.461; top quartile, MTS > −0.322). GSA data were used. For each SNP, we performed a Fisher’s exact test to assess the difference in genotype between top and bottom quartile individuals. A genome-wide significance threshold of 7.69 × 10^−8^ was used, with a suggestive significance threshold of 10^−5^.

### Sensitivity analysis

To measure how independent the identified MTS was from various clinical measures: age, sex, group of origin, disease duration, age at blood collection, MS subtype, severity [through age-related MS severity score (ARMSS)], and type of interferon treatment, we performed a sensitivity analysis. For continuous variables (age, severity, and disease duration), we performed a Pearson’s correlation test. For categorical variables, we performed a Mann–Whitney’s test.

## Results

### DNAm differences and methylation treatment score

**Figure 1:**
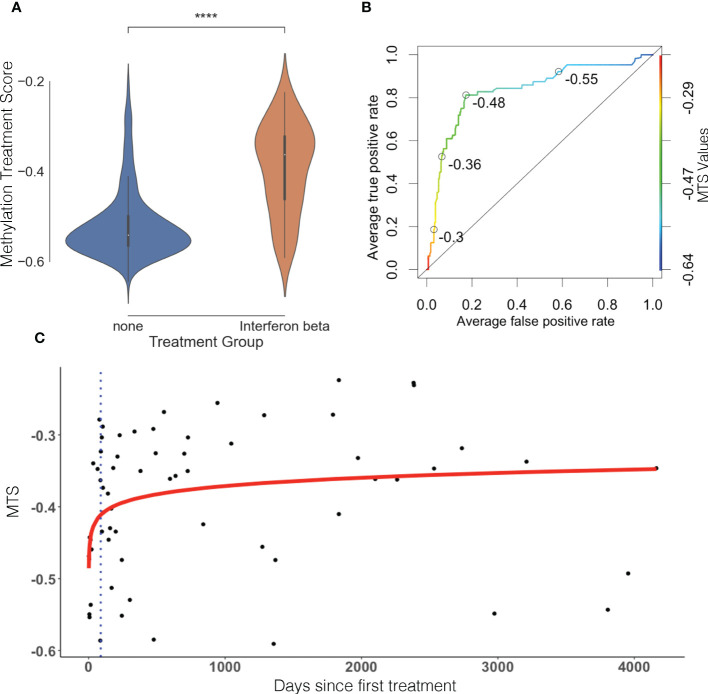
Methylation treatment score can discriminate between treated and untreated samples and is time sensitive. **(A)** MTS is significantly different between treated and untreated patients. Violin plot represents distribution of the MTS. Blue is untreated, and orange is treated. **(B)** Receiver operating characteristic (ROC) curve, AUC = 0.83. Open circles represent various MTS thresholds. Color gradient scale represents MTS values. **(C)** MTS is time-sensitive and reflects therapeutic lag in treated patients. Blue dots represent individual treated samples. Y-axis represents days since first treatment, and X-axis represents MTS. Red line represents line of best fit (p = 9.29 × 10^−3^, R^2 = ^0.104). Blue dotted line represents average therapeutic lag for IFNβ treatment (88.2 days) as defined by Roos et al. (23).

In total, we identified 22 DMPs that were very strongly associated with treatment in both the discovery and replication groups with Δβ differences ranging from around 5% to around 25% (p< 9.8 × 10^−8^ in both cohorts). Using the 11 index DMPs (**Table 2**) and their associated Δβ, we constructed an MTS for each sample, which is a composite score reflecting methylation at all identified CpGs affected by IFNβ treatment. The MTS was strongly associated with treatment status (mean_treated_ = −0.39 ± 0.099, mean_untreated_ = −0.52 ± 0.075, p = 1.1 × 10^−25^, **Figure 1A**; **Supplementary Table 1**). Using an area under the curve (AUC) analysis, we also demonstrated that the MTS accurately discriminates between treated and untreated samples (AUC = 0.83) (**Figure 1B**).

**Table 2:** Index DMPs identified in the discovery dataset and replicated in the replication group.

CpG	Position				Discovery (n = 114)				Replication (n = 235)				Combined p-value
	Chr	Position	Gene	Feature	Mean (treated)	Mean (untreated)	Delta Beta	P-Value	Mean (treated)	Mean (untreated)	Delta Beta	P-value	Combined p-value
**cg01028142**	2	7004578	*CMPK2*	Body	0.77	0.86	−0.09	8.73E-14	0.75	0.83	−0.08	3.79E-15	3.31E-28
**cg24678928**	4	169240829	*DDX60*	TSS1500	0.70	0.80	−0.10	1.52E-09	0.70	0.79	−0.09	9.35E-12	1.42E-20
**cg13452062**	1	79088559	*IFI44L*	5’UTR	0.50	0.77	−0.27	2.06E-10	0.53	0.77	−0.23	2.11E-13	4.34E-23
**cg08888522**	2	163172908	*IFIH1*	Body	0.81	0.89	−0.07	1.60E-10	0.83	0.88	−0.05	4.29E-10	6.86E-20
**cg05552874**	10	91153143	*IFIT1*	Body	0.46	0.57	−0.11	3.00E-10	0.49	0.54	−0.05	3.08E-05	9.25E-15
**cg06188083**	10	91093005	*IFIT3*	Body	0.36	0.45	−0.08	3.39E-08	0.35	0.43	−0.08	4.13E-11	1.40E-18
**cg26312951**	21	42797847	*MX1*	TSS200;5’UTR	0.20	0.28	−0.08	1.25E-10	0.19	0.27	−0.08	2.50E-13	3.12E-23
**cg26505274**	12	121474114	*OASL*	Body	0.72	0.63	0.09	2.45E-10	0.69	0.60	0.09	2.33E-10	5.71E-20
**cg22930808**	3	122281881	*PARP9*	5’UTR	0.49	0.61	−0.12	6.06E-09	0.48	0.58	−0.10	2.81E-10	1.70E-18
**cg06981309**	3	146260954	*PLSCR1*	5’UTR	0.29	0.38	−0.09	3.43E-09	0.30	0.34	−0.04	2.20E-05	7.55E-14
**cg10549986**	2	7018153	*RSAD2*	1stExon	0.11	0.18	−0.07	4.17E-08	0.14	0.20	−0.06	1.88E-09	7.83E-17

Index DMPs were used to construct the MTS.

The MTS in the treated sample group had a larger distribution (wider spread of the data) than the untreated sample group (**Figure 1A**). To assess whether this was caused by genetic determinants and followed a pattern of responder vs. non-responder, we performed a GWAS of the MTS between the top and bottom quartiles individuals based on MTS. This revealed no significant or suggestive differences in genotype at tested loci between treated samples in the top quartile vs. bottom quartile (see **Supplementary Figure 1**), indicating no influence of genotype in response to IFNβ treatment. We therefore explored the relationship between MTS and the day of first treatment. We identified that MTS is associated with the log of days since first treatment (p = 9.29 × 10^−3^, R^2 ^= 0.104; **Figure 1C**). The line of best fit reaches a plateau after around 100 days after first injection. Limiting our modeling to pwMS tested within 100 days of first injection (n = 16) showed increased R^2^ (from 0.104 to 0.2371) while remaining significant (p = 0.0321) (see **Supplementary Figure 2**). This suggests that MTS correlates early with first treatment date when removing pwMS who might be off treatment for long periods of time. Finally, we performed a sensitivity analysis to test between the association of MTS and various covariates. MTS was not associated with age at blood collection, disease severity (measured through ARMSS), disease duration, MS subtype, cohort of origin (discovery vs. replication), or type of treatment (IFNβ 1a vs. IFNβ 1b) (**Supplementary Figure 3**).

### Combined groups overrepresentation analysis

**Figure 2:**
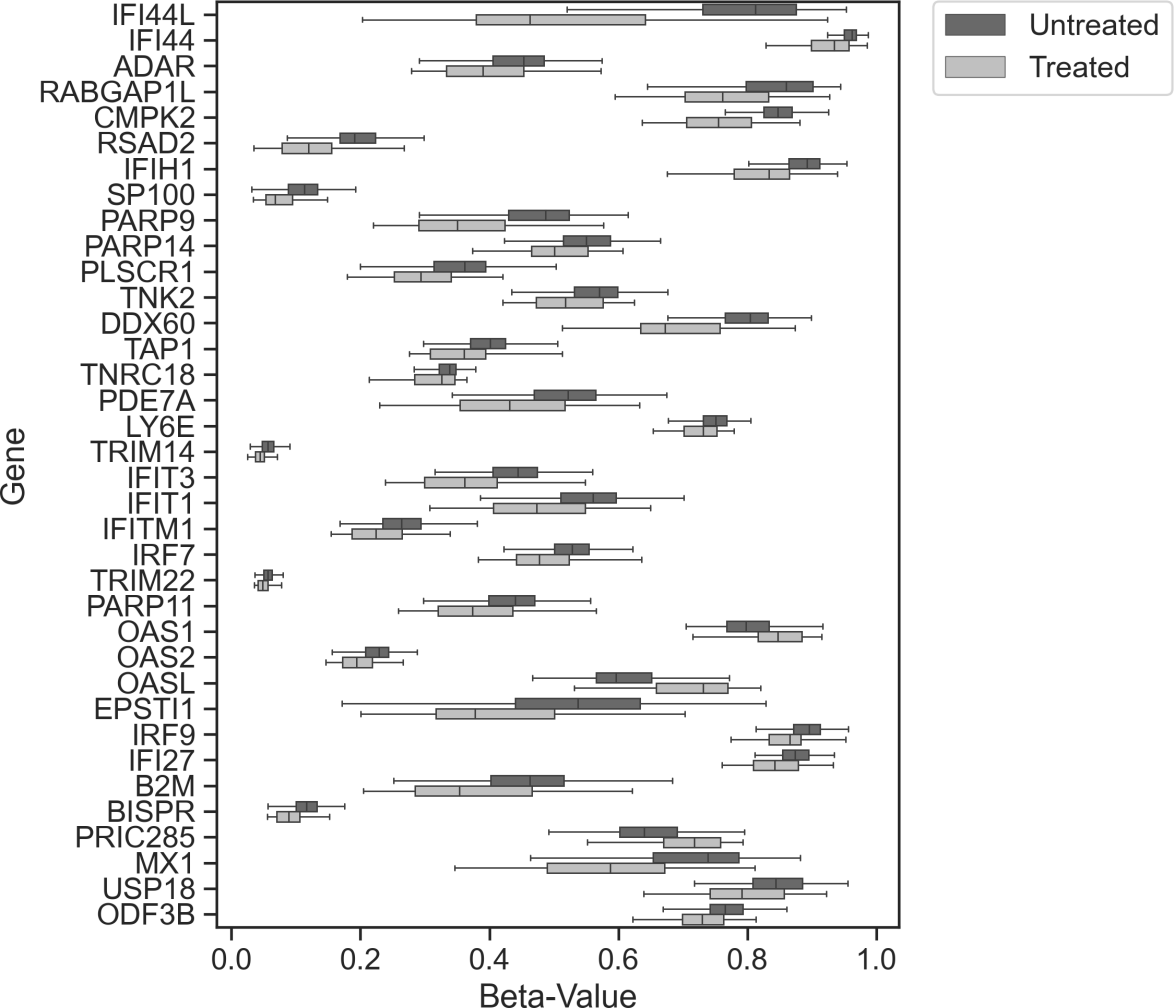
Combined study analysis. Tukey box plot showing the distribution of beta values between treated (light gray) and untreated (dark gray) samples (all test reached p ≤ 9.8 × 10^−8^) for each index DMP associated with each gene.

To determine whether increased statistical power would identify smaller effect size DMPs associated with IFNβ treatment, we conducted an EWAS after combining studies (n = 349). We identified 81 DMPs mapping to 36 different genes (**Figure 2**; **Supplementary Table 2**). With the increased sample size, we identified 59 novel DMPs at 25 additional genes. Twenty-one of the 36 genes (58.3%) had a DMP identified within either the transcription starting site (within 1,500 bp) or in the 5’untranslated region (5’UTR). These DMPs are very likely to modulate the expression of the gene they are mapped to.

**Figure 3:**
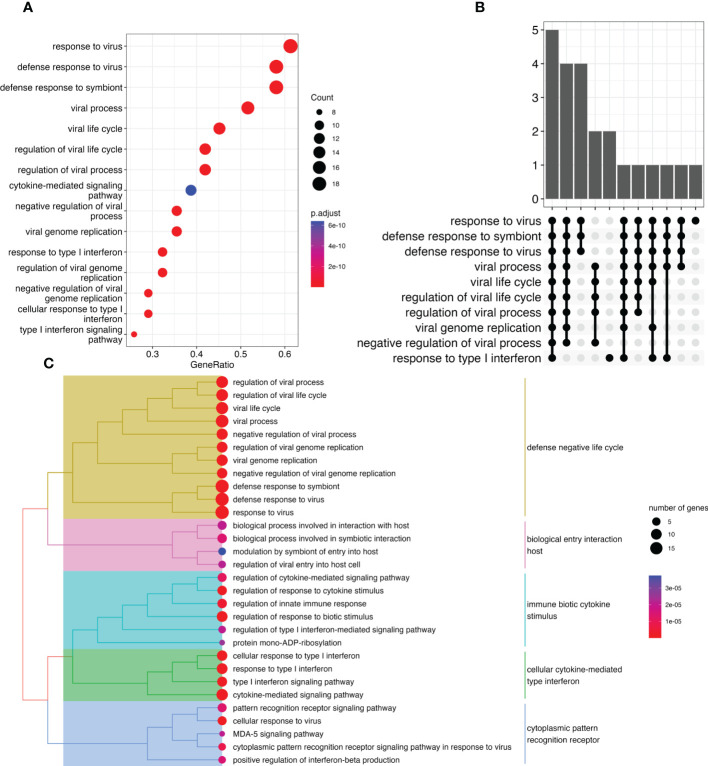
Overrepresentation analysis of genes modulated by IFN treatment reveals virus related response. **(A)** Dot plot representing pathways disrupted between treated and untreated pwMS Y-axis represents pathways identified. X-axis represents the gene ratio (number of entities in gene list vs. number of entities in the pathway) for a specific pathway. Marker size represents the number of entities. Colors represent significance. **(B)** UpSet plot showing the number of genes identified in the ORA for each identified function. Each bar represents a group of gene with the Y-axis representing the number of genes. The dots underneath show which pathways are associated with this group of genes. **(C)** Tree plot representing various differentially methylated pathways grouped by similarity. Dot size represents number of genes, whereas dot colors represent significance. Colors over the hierarchical tree represent pathways sharing similarities.

Using the combined gene list as input for ORA (**Figures 3A–C**) revealed that most of the genes containing DMPs were involved in anti-viral and response to virus infection pathways.

### Cell-specific analysis

**Figure 4:**
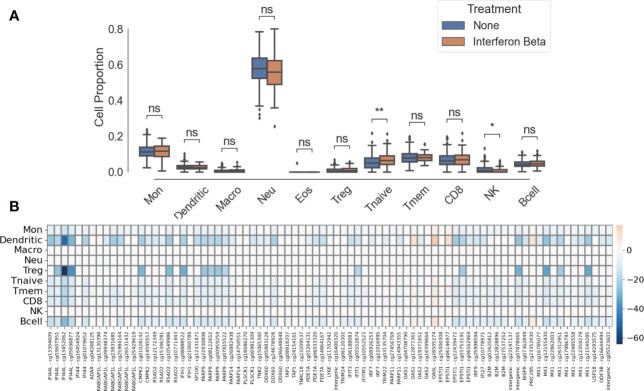
Cell-specific differences between IFNβ-treated patient and untreated patients. **(A)** Immune cell proportions. ns, not significant; *, p< 0.05; **, p< 0.01. **(B)** Heatmap of cell-specific methylation differences between IFNβ-treated and untreated groups. Intensity of colors represents effect size, blue represents hypomethylation, and red represents hypermethylation. CpGs labeled “intergenic” are not mapped to a gene.

One limitation of whole blood EWAS is the bulk analysis of different immune cell types. To overcome this limitation, we performed immune cell deconvolution through a two-step process. First, we estimated the proportion of each of 11 immune cell types (**Figure 4A**). Second, using multiple linear regression (see Methods), we performed statistical deconvolution to identify methylation differences between treated and untreated patients in each immune cell type (**Figure 4B**). Cell proportions were not statistically significantly different between in treated and untreated pwMS except for NK cells (mean_treated_ = 0.010, mean_untreated_ = 0.016, p< 0.01) and CD4^+^ naïve T cells (mean_treated_ = 0.071, mean_untreated_ = 0.053, p< 0.05). We identified the largest methylation differences in regulatory CD4^+^ T cells (Tregs) with 16 cell-specific DMPs and dendritic cells (DCs) with 53 cell-specific DMPs (**Figure 4B**). Smaller changes can be observed in monocytes, B cells (55 cell-specific DMPs), and other T cell subsets (**Figure 4B**; **Supplementary Table 3**). No significant methylation differences were detected in NK cells, neutrophils, or macrophages. The gene with the biggest methylation difference is *IFI44L* in both Treg and DCs.

## Discussion

The role of endogenous IFNβ during a viral infection is protective, promoting the activation of antigen-presenting cells and subsequent expansion of T and B cells (24). Here, we have demonstrated that that IFNβ treatment is associated with modification of specific DNAm sites in a time-dependent manner.

We found that DNAm changes after treatment are specific to interferon genes and are reproducible across samples. Methylation changes after IFNβ can be summarized into an MTS that has strong discriminating power. In addition, this MTS is impervious to many parameters, such as age, disease duration, severity, or MS subtype. Roos and colleagues investigated, in a 2020 MSBase data study the therapeutic lag, the delay between first treatment and reaching treatment efficacy, associated with various MS treatments (IFNβ 1b, IFNβ 1a subcutaneous, IFNβ 1a intramuscular, alemtuzumab, natalizumab, mitoxantrone, fingolimod, dimethyl fumarate, teriflunomide, and glatiramer acetate) (23). They identified a range of delayed efficacy ranging from 12.6 weeks (or 88.2 days) for IFNβ 1b to 27.5 weeks for dimethyl fumarate. Interestingly, the identified lag after commencement of INFβ coincides with the inflexion point of the best fit line between day of first treatment and MTS. Therefore, our MTS is a potential treatment efficacy biomarker. The fact that the previously identified therapeutic lag and methylation changes are strongly associated indicates that methylation modification is reflecting IFNβ treatment efficacy. However, this needs to be experimentally confirmed. We did not have information about the date of the last dose and were therefore unable to investigate the reversibility of the methylation effect from IFNβ treatment. Some samples have a similar MTS to those of untreated samples, potentially indicating that treatment might have stopped. A follow-up analysis, focusing on the reversibility of methylation changes, will be of particular interest for pwMS wishing to switch to other treatment, as methylation changes might affect other treatment’s efficacy.

Genes with modified methylation profiles were all known interferon response genes that are involved in response to viral pathways. This could be because IFNβ recruits endogenous anti-viral machinery and provokes long-lasting changes in those genes through methylation. The gene with the widest gap in beta value between treated and untreated pwMS (both in whole blood and deconvoluted cell analysis) is *IFI44L*, which is known to modulate virus replication and anti-viral state (25).

We have also provided evidence that there are large effect DNAm changes in both DCs and Treg cells, with smaller effects identified in monocytes, all CD4^+^ T cells subsets, CD8^+^ T cells, and B cells. IFNβ treatment plays a significant role in the activation and migration of DCs, modulating the activation of downstream T cell subpopulations (26). Importantly, DCs induce proliferation of regulatory T cells following IFNβ treatment (27). Increase in CD4^+^ T cell expansion and survival can be observed in acute viral infections when endogenous IFNβ is released (28). This is similar to the increase that we observed in the CD4^+^ T naïve cell population in IFNβ-treated patients. The absence of methylation changes identified in neutrophils, NK cells, and macrophages suggests that any innate immunity response emerging from IFNβ treatment is not mediated through methylation, as shown by the difference of NK proportion between treated and untreated patients. The IFNβ treatment effect on DNAm identified in this study is unique and different compared to that described after dimethyl fumarate (7) or mixed DMT treatment (29). Because of the historic nature of these whole blood samples, we were unable to validate our findings in isolated cell types. However, the statistical deconvolution analysis methods for whole blood, particularly the reference-based algorithms such as what we have used here, are now excellent at replicating the isolated cell type results. It is a convenient way to investigate cells that are found in low proportions in the peripheral blood and are difficult to obtain enough material from for analysis.

Although IFNβ treatment has widely been superseded by higher efficacy treatment in the management of MS, it remains relevant in 2023. IFNβ treatment activates anti-viral response and specifically depletes memory B cells (30, 31). Memory B cells are the known reservoir of Epstein–Barr virus (EBV) persistence, a virus that has been recently highlighted as the strongest risk factor for MS (32). Depleting this specific B memory population could help reduce the probability of EBV reactivation. In addition, through anti-viral activity, IFNβ treatment was proposed as a protective treatment for SARS-CoV-2 infections compared to other high efficacy treatments (33, 34), but later studies reported limited to no efficacy (35). Finally, there is a wide body of work suggesting that vitamin D has an effect of MS risk. Although vitamin D supplementation alone does not seem to influence MS progression and severity in pwMS (36–39), IFNβ and vitamin D combination could remain relevant as vitamin D enhances IFNβ response (40).

## Conclusion

**Figure 5:**
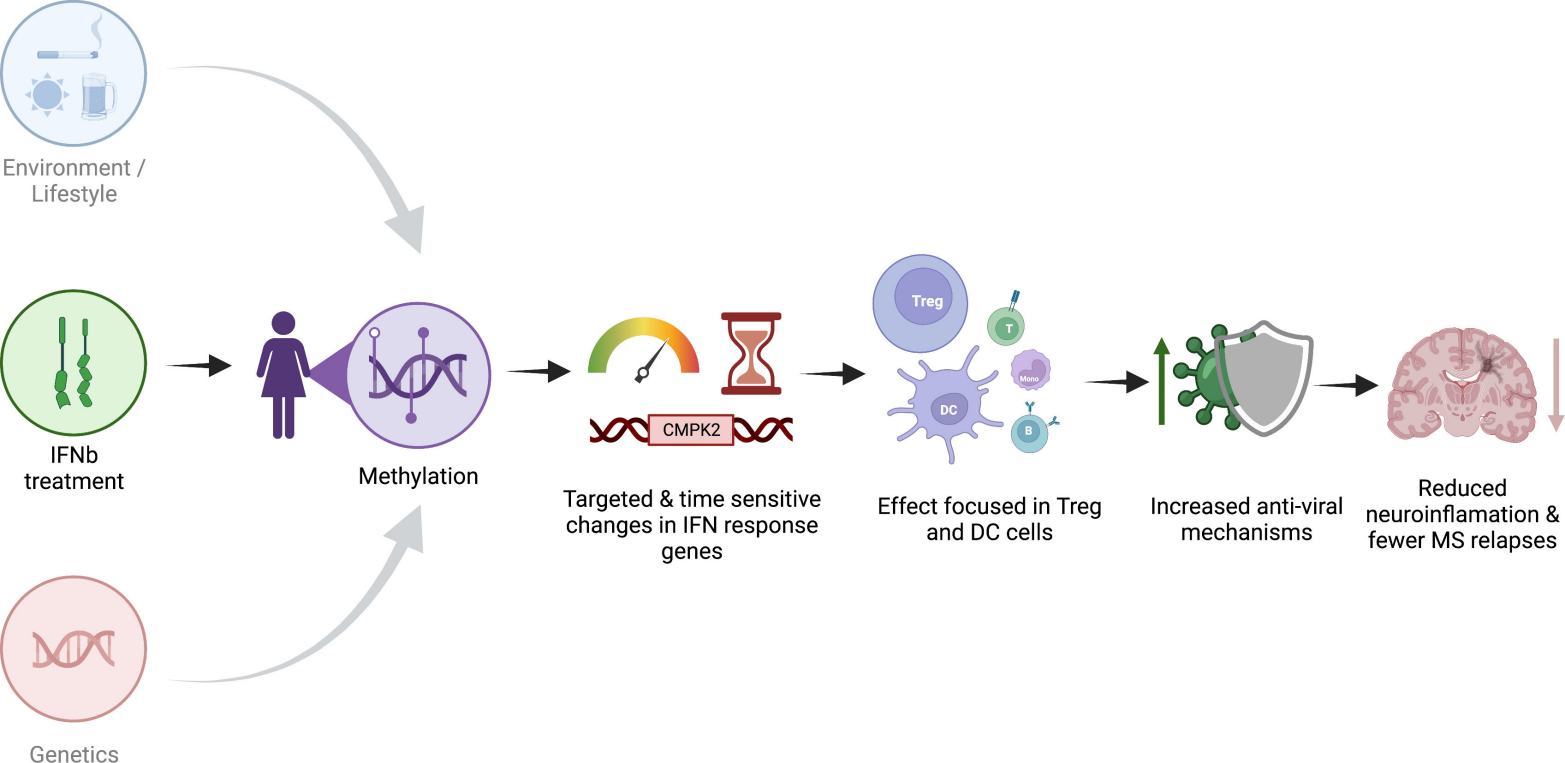
Schematic representation of the proposed effect of IFNβ treatment on methylation. Created with biorender.com.

Together, our results suggest that IFNβ treatment has a strong, targeted, and reproducible effect on DNAm in MS patients (**Figure 5**). The association of our MTS with treatment start suggests that treatment efficacy is at least partially associated with methylation changes and that changes in MTS might reflect response to treatment. An MTS that reflects response to treatment may be of interest for clinicians because a change in MTS could indicate non-response to treatment and the need to change to a different treatment. Our MTS is also a convenient way to assign treatment response for researchers performing methylation studies and needing to correct for the influence of IFNβ treatment. The association between MTS and treatment date suggests that treatment efficacy is at least partially associated with methylation changes.

The pathways that we identified as modulated by IFNβ treatment suggest that IFNβ treatment most likely recruits viral infection response pathways with highly targeted, cell-specific effects to reduce overall inflammation, resulting in reduced relapses and disease activity.

## Data availability statement

The datasets presented in this article are not readily available because of the General Data Protection Regulation as well as study-specific ethics and governance terms and conditions. Requests to access the datasets should be directed to JL-S (Jeannette.LechnerScott@health.nsw.gov.au).

## Ethics statement

All participants to this study gave their written informed consent. The Ausimmune Study was approved by nine regional Human Research Ethics Committees (study number 09/04/15/5.04 and HREC/09/HNE/139). The patients/participants provided their written informed consent to participate in this study.

## Author contributions

RS, JL-S, VGJ, and RL contributed to the conception of the work. RS, JS, VGJ, and RL contributed to the design of the work. AX, MC, TK, BT, A-LP, RS, JL-S, VGJ, and RL contributed to the acquisition of data. AX, MC, VM, and RL contributed to the analysis of data. AX, MC, VM, JL-S, and RL contributed to the interpretation of data. AX, VM, RS, JL-S, and RL contributed to the manuscript drafting. VM, TK, BT, AP, RS, JL-S, VGJ, and RL contributed to the manuscript substantial revision.

## Supplementary material

Supplementary Figure 1 Manhattan plot showing genome-wide association between genotype and MTS. Genotype was compared between high-MTS (top quartile) and low-MTS (lowest quartile) pwMS. Each dot represents -log10(p) with a genome-wide significance set at 7 (or -log10(10e-8)). Alternating shades of grey represent chromosomes.

Supplementary Figure 2 Regression showing association between days of first treatment and MTS, only with pwMS whose blood was collected within the first 100 days of treatment. Each dot represents a sample, and the red line is the line of best fit (MTS = -0.54 + 0.035 * log10(Days since first treatment)). p = 0.032, R2 = 0.237.

Supplementary Figure 3 Sensitivity analysis of MTS against several covariates. a) MTS vs MS course (CIS = Clinically isolated syndrome, RR = relapse remitting, PP = primary progressive, SP = secondary progressive). No significance between untreated samples from different MS courses. No significance between treated samples from different MS courses b) MTS vs Study group c) MTS vs age d) MTS vs Sex e) MTS vs ARMSSS (Age relates MS Severity Score) f) MTS vs Disease durations g) MTS vs Type of interferon. r = Pearson’s correlation coefficient, p = p-value of pearon’s correlation test, ns = non significant or p > 0.05, * p< 0.05, ** p< 0.01, *** p<0.001, ****p<0.0001
