# Supplementary figures and images for: Interferon beta treatment is a potent and targeted epigenetic modifier in multiple sclerosis
Source: Front Immunol. 2023 May 30;14:1162796. doi: 10.3389/fimmu.2023.1162796 (PMC10266220; doi:10.3389/fimmu.2023.1162796)

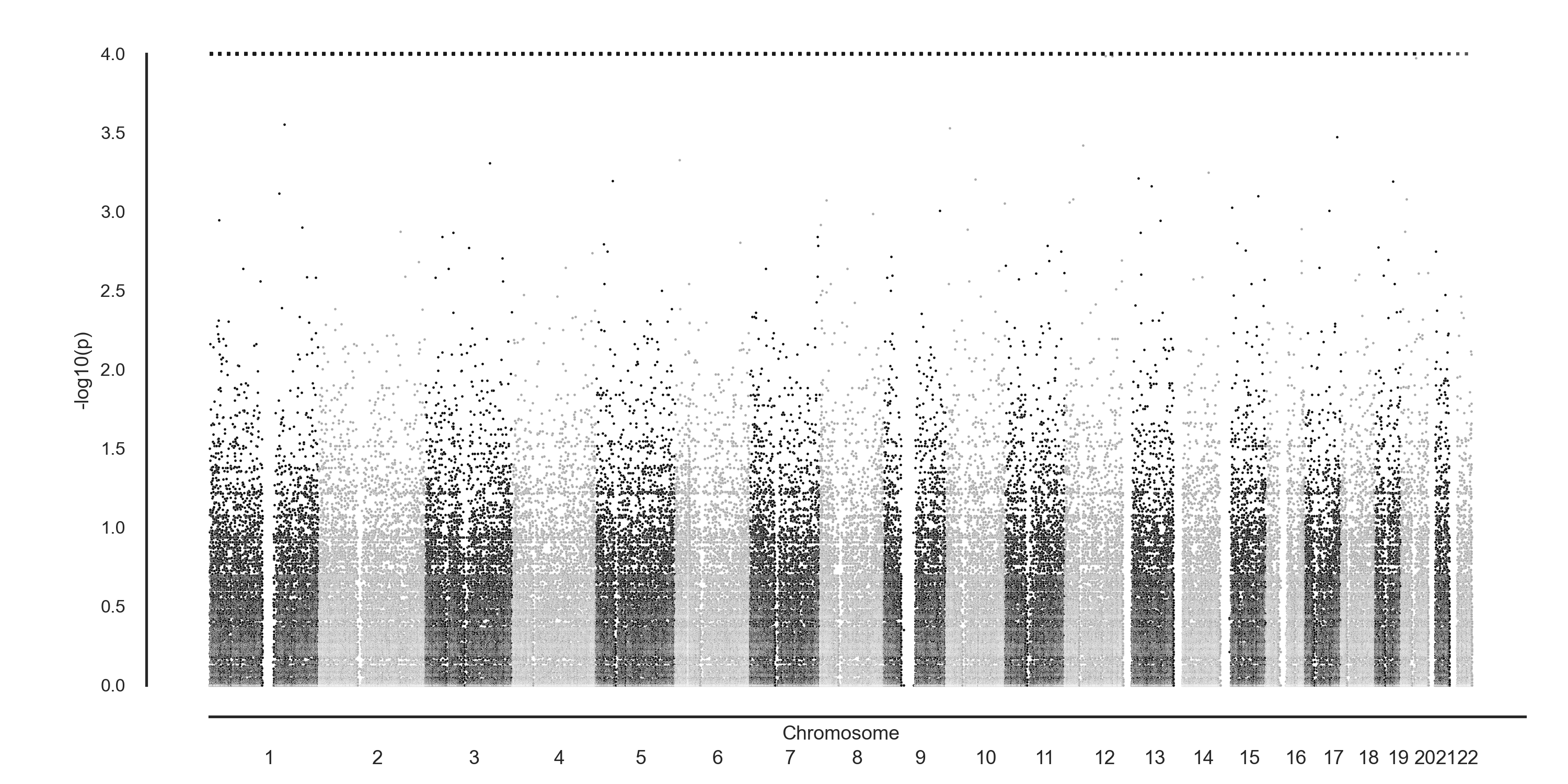

Supplement: Supplementary Figure 1 — Manhattan plot showing genome-wide association between genotype and MTS. Genotype was compared between high-MTS (top quartile) and low-MTS (lowest quartile) pwMS. Each dot represents -log10(p) with a genome-wide significance set at 7 (or -log10(10e-8)). Alternating shades of grey represent chromosomes. [file Image_1.tiff]

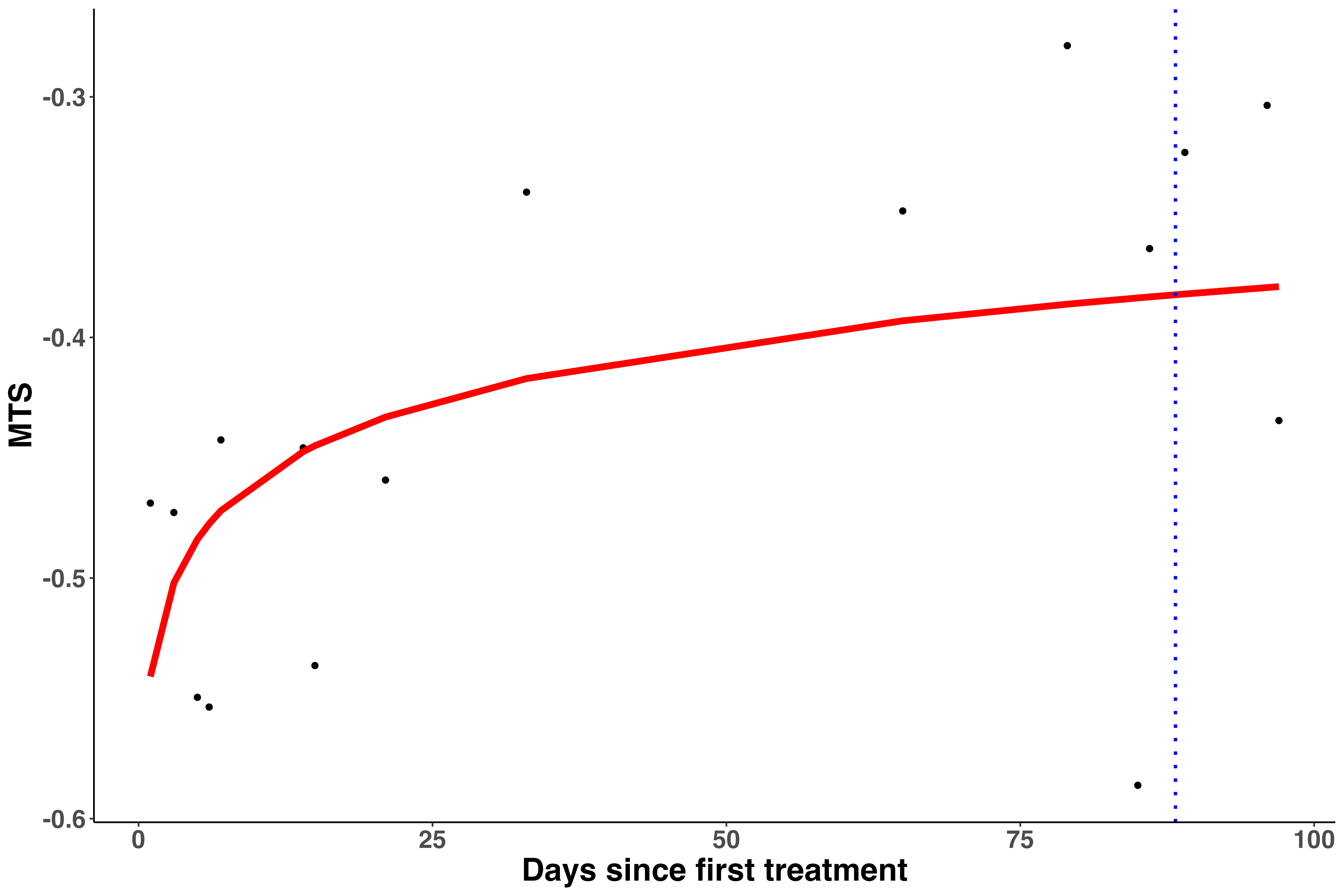

Supplement: Supplementary Figure 2 — Regression showing association between days of first treatment and MTS, only with pwMS whose blood was collected within the first 100 days of treatment. Each dot represents a sample, and the red line is the line of best fit (MTS = -0.54 + 0.035 * log10(Days since first treatment)). p = 0.032, R2 = 0.237. [file Image_2.tiff]

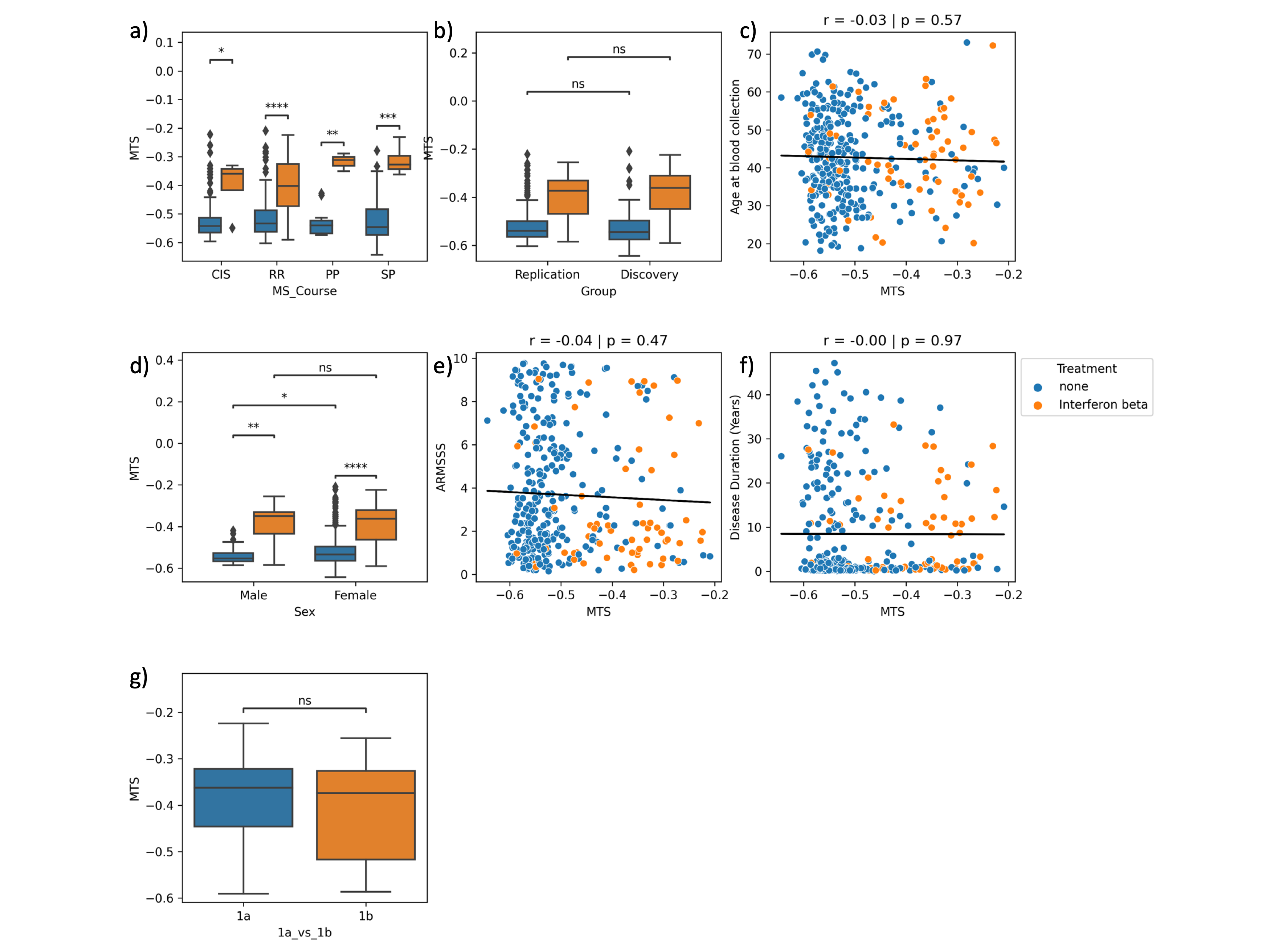

Supplement: Supplementary Figure 3 — Sensitivity analysis of MTS against several covariates. a) MTS vs MS course (CIS = Clinically isolated syndrome, RR = relapse remitting, PP = primary progressive, SP = secondary progressive). No significance between untreated samples from different MS courses. No significance between treated samples from different MS courses b) MTS vs Study group c) MTS vs age d) MTS vs Sex e) MTS vs ARMSSS (Age relates MS Severity Score) f) MTS vs Disease durations g) MTS vs Type of interferon. r = Pearson’s correlation coefficient, p = p-value of pearon’s correlation test, ns = non significant or p > 0.05, * p< 0.05, ** p< 0.01, *** p<0.001, ****p<0.0001 [file Image_3.tiff]
